# 

**Published:** 1883-04

**Authors:** 


### CONTENTS:

ART. I.—Are Dentists' Exempt from Jury Duty? .......................529

ART. II.—The Hygienic Relations of Artificial Dentures By G A. D. G........542

ART. III—Odonto-Chirurgical Society of London England.....................544

ART. IV.—Diseases of the Dental Pulp. By Dr. Samuel A. White..............557

ART. V.—Oxy-Phosphate of Zinc. By E. G. Betty, D. D. S....................564

South Carolina State Dental Association....................568

Texas State Dental Association....................569

Dental Society of the State of New York....................569

### EDITORIAL, ETC.

University of Maryland, Dental Department, Annual Commencement ............5[illegible]0

Alumni Association of the Dental Department of the University of Maryland .....572

A Useful Invention.......................................572

### MONTHLY SUMMARY.

Teeth Injured by Tobacco....................573

Separation of Silver From Alloys....................574

A Conclusion not Warranted by Facts....................574

Gold Alloy for Solder....................575

Filling Over-Exposed Nerves....................575

A Dentists' Wife's Death....................576

